# Piezoelectric Response and Cycling Fatigue Resistance of Low-Temperature Sintered PZT-Based Ceramics

**DOI:** 10.3390/ma16041679

**Published:** 2023-02-17

**Authors:** Zirui Lin, Zhe Zhu, Zhonghua Yao, Hao Zhang, Hua Hao, Minghe Cao, Hanxing Liu

**Affiliations:** 1 School of Material Science and Engineering, Wuhan University of Technology, Wuhan 430070, China; 2 Sanya Science and Education Innovation Park of Wuhan University of Technology, Wuhan 430070, China

**Keywords:** ceramics, sintering aids, piezoelectric ceramics, multilayer

## Abstract

The preparation of low-cost multilayer piezoelectric devices requires using cheap internal electrodes between the dielectric layers. A general strategy is to reduce the sintering temperature *T*_s_ of the ceramic layer by sintering aids which can form a liquid phase. Here, 0.2 wt% Li_2_CO_3_ was added as a sintering aid to tailor the sinterability and piezoelectricity of the commercial PZT ceramics. As verified from experiments, the piezoelectric ceramics could be densified at a sintering temperature above 940 °C, suitable for co-firing with the cheap internal electrode. The optimized sintering temperature of 980 °C can be confirmed for the 0.2 wt% Li_2_CO_3_-modified PZT ceramics due to its high piezoelectric coefficient *d*_33_ ~ 701 pC/N, planar coupling factor *k*_p_ ~ 66.7%, and a low mechanical quality factor *Q*_m_ ~ 71 with a transition temperature of *T*_c_ ~ 226 °C, presenting the characteristics of typical soft piezoelectric ceramics. Moreover, both the potential piezoelectric strain ~0.13% under 20 kV/cm and the good cycling fatigue characteristic (>10^4^ cycles) of the studied piezo compositions indicates strong competitiveness in the field of multilayer piezoelectric devices.

## 1. Introduction

Piezoceramics come from the general design of ferroelectrics due to the piezoelectric effect via electric-field-driven polarization treatment of polycrystals. These piezoelectric effects can generate an electro-response or micro-displacement response, which can be used for electronic components and devices for displacement and electrical-signal sensitivity [1,2,3]. Compared to electromagnetic actuators, piezoelectric ceramics mainly feature compact size, low driving voltage, quick response, high efficiency, accurate position, etc. [4,5,6,7].

Chemical doping dominates the structural design of piezoelectric materials to form donor-type (soft) or acceptor-type (hard) characteristics. Doping high-valence ions (e.g., La^3+^, Nd^3+^, Sm^3+^, Nb^5+^ and Ta^5+^) always cause lead vacancies, which is beneficial to domain rotation during electric field polarization, and further improve dielectric, electromechanical, and piezoelectric properties, similar to softening ceramics [8,9,10,11]. Soft piezoelectric ceramics are characterized by high direct piezoelectric constant *d*_33_, strain rate *S*, significant remnant polarization *P*_r_, and low coercive field *E*_c_. Typical soft Pb-based compositions by La doping, Pb_1−x_La_x_(Zr_y_Ti_1−y_)_1−x/4_O_3_ (abbreviated as PLZT) ceramics have been extensively studied due to their high electromechanical and electro-optical properties. La/Zr/Ti ratio can make the PLZT ceramics own various phase structures such as ferroelectric, relaxor ferroelectric, antiferroelectric, and paraelectric phases for diverse applications. Wu et al. reported Pb_0.91_La_0.06_(Zr_0.58_Ti_0.42_)_0.975_Nb_0.02_O_3_ prepared via the ceramic injection molding (CIM) technique with ultra-high *d*_33_ (731 pC/N) [12]. Kumar et al. reported a high effective piezoelectric constant *d*_33_^*^ ~ 632 pm/V and a high unipolar strain rate *S* ~ 0.25% in Pb_0.92_La_0.08_(Zr_0.60_Ti_0.40_)O_3_, accompanied by a high *P*_r_ ~ 33 µC/cm^2^ and a low *E*_c_ ~ 10 kV [13]. As reported, Nb doping can compensate for the vacancy at the B site for ABO_3_-type perovskites caused by La doping. Nb-doping also has been proven to contribute to the improvement of piezoelectric responses and fatigue behavior, then the decrease in the coercive field [14,15]. In addition, Nb oxide is helpful to densify the Pb(Zr_1−x_Ti_x_)O_3_ (abbreviated as PZT)-based piezoceramics and decrease the sintering temperature slightly [14].

A multilayered design can be instrumental in the increase in micrometric displacement and the reduction in the driving field of PZT-based piezoelectric devices [16]. Multilayer ceramics are designed to co-sinter the inner electrodes and tape-cast ceramic sheet layers together. Developing the multilayered design is essential to lower the cost of products via co-sintering ceramic layers with cheap electrode paste, such as pure Ag or high Ag/low Pd paste instead of expensive platinum (Pt)/palladium (Pd) electrode paste. However, the low-temperature stability of cheap metal electrode paste limits the co-sintering of traditional PZT-based systems due to high sintering temperatures above 1200 °C [17,18]. There are various strategies to lower the sintering temperature of PZT-based piezoceramics, such as nanosized powder sintering [19], liquid phase assisted sintering [20], cold sintering process [21], high-energy ball milling [22], etc. Among these, the most straightforward and economical method is to form a liquid phase by sintering aid. Metal oxides/carbonates, such as B_2_O_3_, CuO, Bi_2_O_3_, ZnO, and Li_2_CO_3_, are the most used sintering additives. Moreover, the complex sintering aids can make low-temperature sintering effective [23]. Zhang et al. confirmed that the combined use of LiBiO_2_ and CuO has an excellent enhancement effect on *d*_33_^*^ and optimal sinterability compared to pure LiBiO_2_ [24]. Choi et al. investigated that the mixed 4PbO-B_2_O_3_ liquid frits could optimize the sintering temperature of 0.4Pb(Zn_1/3_Nb_2/3_)O_3_–0.6Pb(Zr_0.47_Ti_0.53_)O_3_ piezoceramics, providing a liquid phase at below 800 °C [25].

The mechanism of sintering aids is that it can form a liquid transitional phase at a relatively low temperature, which can facilitate the dissolution and migration of substances, accelerate grain growth, and finally be absorbed into the ceramic to form a solid solution in the lattice and impurity at the grain boundary. Donnelly et al. reported that Li_2_CO_3_ reacted with excess PbO to form an intermediate liquid phase, the Li_2_PbO_3_ phase, which subsequently melted at 836 °C, resulting in the promotion of particle rearrangement [26]. Siddiqui et al. studied the effect of Li_2_CO_3_ on Pb_0.93_La_0.02_Sr_0.05_(Zr_0.52_Ti_0.48_)O_3_ (abbreviated as PLSZT) ceramics sintered at 850 °C. They found that the relative permittivity *ε*_r_ ~ 1270 and the planar electromechanical couple factor *k*_p_ ~ 46% were enhanced compared to those without Li_2_CO_3_ sintered at 1150 °C (*ε*_r_ ~ 1145, *k*_p_ ~ 33%) [27]. However, *d*_33_ decreased from 315 pC/N for pure PLSZT to 259 pC/N for the modified one. Kim et al. reported that CuO-doped 0.69Pb(Zr_0.47_Ti_0.53_)O_3_-0.31[Pb(Zn_0.4_Ni_0.6_)_1/3_Nb_2/3_]O_3_ ceramics exhibited a higher strain of 0.165% than that 0.145% for undoped ones [28]. Zhang et al. revealed that Li^+^ could segregate at the grain boundaries and suppress grain growth of 15Pb(Sc_1/2_Nb_1/2_)O_3_-52Pb(Mg_1/3_Nb_2/3_)O_3_-33PbTiO_3_ (abbreviated as PSN-PMN-PT) piezoceramics when excessive Li_2_CO_3_ was employed when sintered at 850 °C [29]. Both *d*_33_ and *k*_p_ increased from 465 pC/N and 60% for pure PSN-PMN-PT ceramics to 520 pC/N and 62% for 0.5 wt% Li_2_CO_3_-modified one, respectively. Some other examples of low-temperature piezoceramics are listed in Table 1. It has been reported that Li_2_CO_3_ is a typical sintering aid usually used in PZT-based ceramics. The effect of the content of sintering aids on lead-based piezoelectric ceramics has been experimentally and theoretically studied. Commonly, the addition of sintering aids can always decrease the sintering temperature at the expense of reducing piezoelectricity. In this work, an appropriate amount of 0.2 wt% Li_2_CO_3_ was chosen as the sintering aid of commercial soft PZT-based piezoceramics to develop the low-temperature cofired piezoelectric ceramic system. The modified ceramics can be cofired with cheap metal electrodes while retaining excellent piezoelectric response.

## 2. Materials and Methods

Commercial soft PZT-based (PZT-5H) piezoelectric powders (Xi’an Konghong New Material Technology Co., Ltd., Xi’an, China) were selected as the base materials, and the Li_2_CO_3_ (99% purity, Shanghai Aladdin Reagent Co., Ltd., Shanghai, China) was selected as the sintering aid. The base materials were designed to form the 0.2 wt% Li_2_CO_3_-modified compositions for fabricating low-temperature cofired ceramic devices. The mixed powders were milled with ethanol for one day. The calcination process was conducted at 850 °C for 2 h. The resultant powders were granulated with a 2.0 wt.% polyvinyl alcohol (PVA) aqueous solution. Pressing was carried out to form the disc samples 12 mm in diameter under 180 MPa. The binders were fired at 600 °C for 2 h, and then the ceramics were sintered from 900 °C to 1100 °C for 2 h.

The ceramics were ground and polished up to a thickness of 0.5 mm on sandpaper. Density measurement was conducted using the Archimedes method. Silver pastes with conducting compositions of 80% Ag and 3% Pd were coated and fired for 10 min at 580 °C on both sides of the samples. The poling of the samples was conducted in silicon oil at 120 °C for 30 min under 25 kV/cm. The ceramics were aged for one day before piezoelectric measurements. The crystal structure was determined by X-ray diffraction (XRD) with *CuK*_α_ = 1.54Å. The microstructures were observed through a scanning electron microscope (SEM, JSM-6700F). The piezoelectric *d*_33_ was measured by a ZJ-3A quasi-static piezoelectric meter 1 pC/N, and 0.2 N. Dielectric property-temperature spectra were characterized by an E4980A LCR meter connected with the temperature control system (Partulab, DMS-1000) in the temperature range from RT to 500 °C at a heating rate of 2 °C/min. The *S-E* relations can be evaluated by a ferroelectric workstation (aixACCT, TF analyzer 2000) under 20 kV/cm electric field strength. The polarization-filed (*P-E*) hysteresis loops were determined by the ferroelectric analyzer (PolyK, PK-CPE1701). The impedance *|Z|* and phase angle *θ* were collected by the impedance analyzer (Agilent, HP4294A, Santa Clara, CA, USA). The *k*_p_ value was calculated according to Equation (1) [37]:

(1) $$k_{p}=\frac{1}{\sqrt{0.395\;\frac{f_{r}}{f_{a}-f_{r}}+0.574}}$$

where f_r_ and f_a_ represent resonance and antiresonance frequencies, respectively, the mechanical quality factor Q_m_ was calculated by Equation (2):

(2) $$Q_{m}=\frac{f_{a}^{2}}{2\;\pi \;f_{r}\;R\;\left(c_{0}+c_{1}\right)\;\left(f_{a}^{2}-f_{r}^{2}\right)}$$

where R, c_1_, and c_0_ are the minimum impedance at resonance, the capacitance of the ceramics at resonance, and the capacitance in an electrostatic field, respectively [37].

## 3. Results and Discussion

### 3.1. XRD Patterns

**Figure 1:**
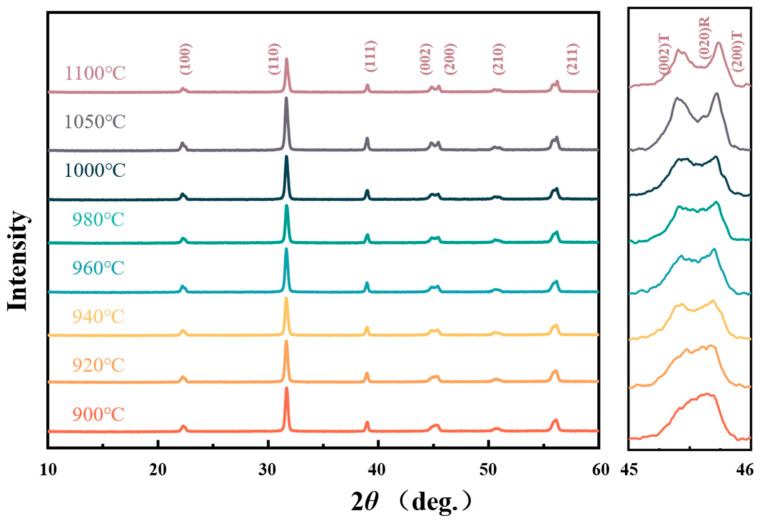
XRD patterns for the 0.2 wt% Li_2_CO_3_-modified bulk ceramics.

Figure 1 presents the phase structure of the sintered 0.2 wt% Li_2_CO_3_-modified bulk ceramics. All of the specimens show the pure perovskite single phase without any impurity composition under the limit of X-ray detection. The broadening of the (200) peak is observed in the samples sintered at 900~1000 °C at the 2*θ* = 44~46° region, which is evidence of the coexistence of the tetragonal and rhombohedral phases. In other words, near the morphotropic phase boundary (MPB). It is generally believed that piezoelectric ceramics have the optimum piezoelectric properties near the MPB. When the sintering temperature increases, a distinct splitting of the (002)_T_ and (200)_T_ peaks can be observed, which implies that the coexistence of the rhombohedral-tetragonal phase near MPB tends to transform into the tetragonal-rich phase. As reported, this transformation can be attributed to the stress relaxation caused by the grain growth of piezoelectric ceramics [38,39]. The ferroelectric ceramics will form an inner stress conducive to the stability of the rhombohedral phase, as reported. However, with the increase of the grain size, the internal stress is challenging to continue to concentrate and finally turns into cracks, releasing this stress and resulting in the rise of tetragonal phases [40].

### 3.2. SEM Images

**Figure 2:**
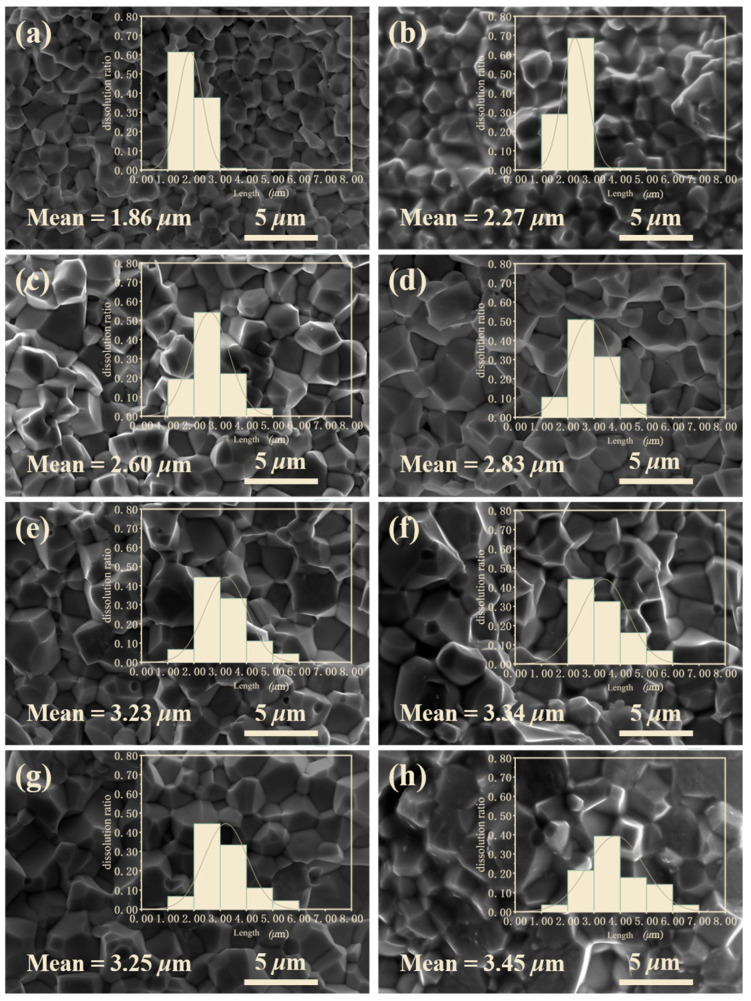
SEM images and grain size distribution for the 0.2 wt% Li_2_CO_3_-modified ceramics sintered at various temperatures: (**a**) 900 °C; (**b**) 920 °C; (**c**) 940 °C; (**d**) 960 °C; (**e**) 980 °C; (**f**) 1000 °C; (**g**) 1050 °C; (**h**) 1100 °C.

Figure 2 shows the fractured cross-sectional SEM images and grain size distribution diagrams of the specimens at different sintering temperatures. Grain grows gradually, consistent with the sintering temperature. The average grain size of ceramic increases from 1.86 μm at 900 °C to 3.45 μm at 1100 °C. It can be observed that grain growth remains constant above 980 °C, with an average grain size of around 3.30 μm, similar to the previous report [31].

**Figure 3:**
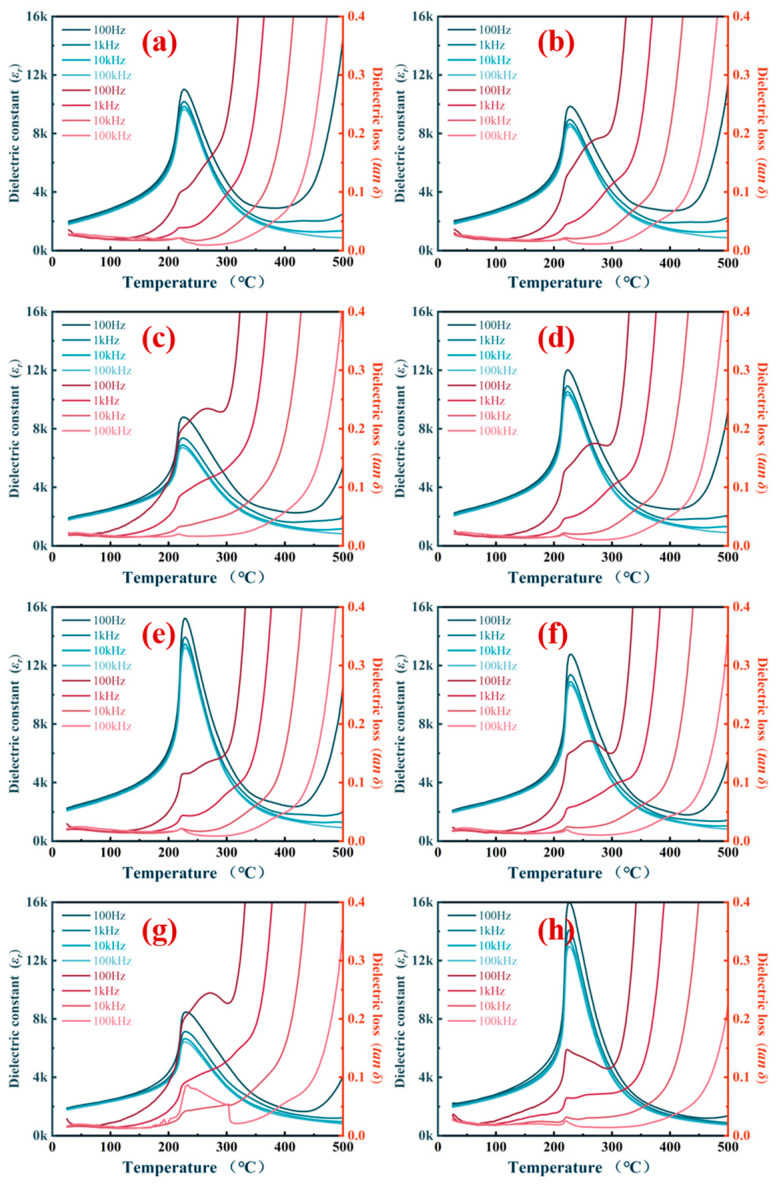
Dielectric property-temperature spectra of the 0.2 wt% Li_2_CO_3_-modified ceramics sintered at different temperatures: (**a**) 900 °C; (**b**) 920 °C; (**c**) 940 °C; (**d**) 960 °C; (**e**) 980 °C; (**f**) 1000 °C; (**g**) 1050 °C; (**h**) 1100 °C.

Figure 3 depicts dielectric property-temperature spectra of the 0.2 wt% Li_2_CO_3_-modified ceramics with various frequencies (100 Hz, 1 kHz, 10 kHz, and 100 kHz). With the increase in testing frequency, no shift or broadening of the dielectric peak was observed. Dielectric maximum peaks show sharp phase transitions indicative of a typical ferroelectric characteristic without any relaxor behavior. It can be concluded that the Curie temperature of the 0.2 wt% Li_2_CO_3_-modified ceramics is around 226 °C. It can be deduced that piezoelectric devices based on these compositions can be used in temperatures above 110 °C. For that, the poled piezoelectric ceramics often begin to become unstable at a temperature somewhat above half the Curie temperature.

**Figure 4:**
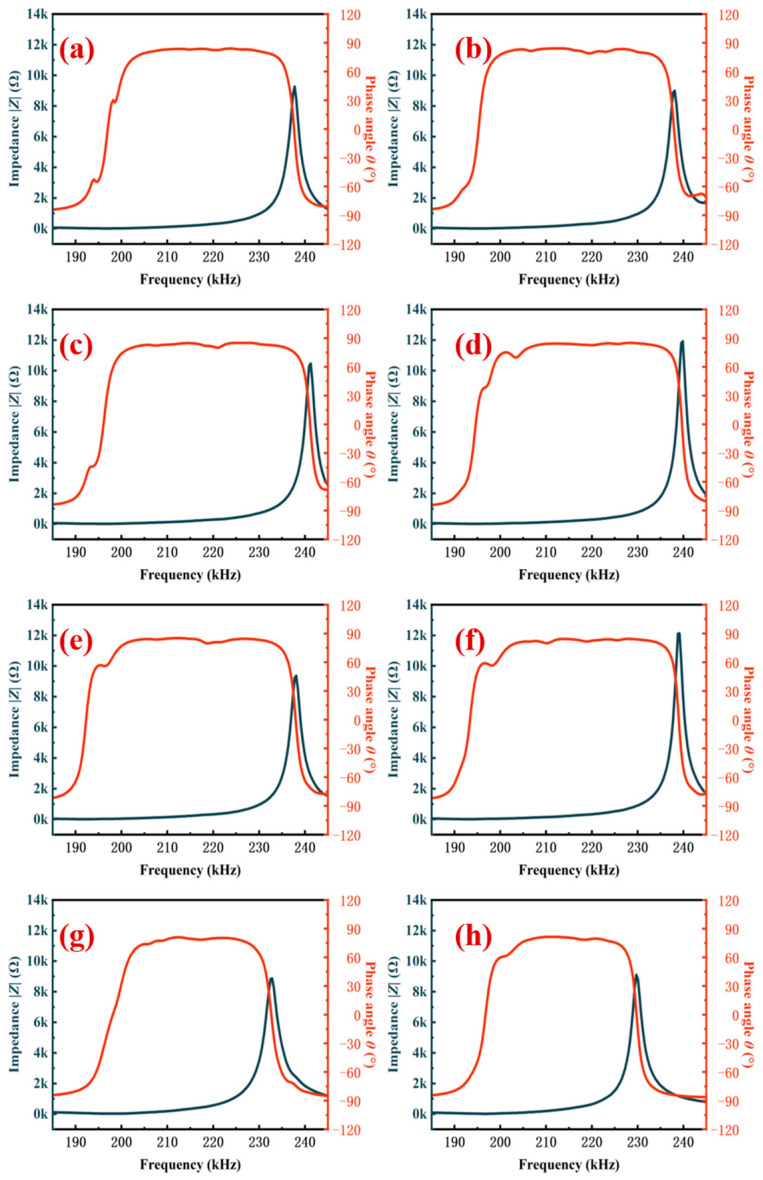
Impedance plots of the 0.2 wt% Li_2_CO_3_-modified ceramics: (**a**) 900 °C sintered; (**b**) 920 °C sintered; (**c**) 940 °C sintered; (**d**) 960 °C sintered; (**e**) 980 °C sintered; (**f**) 1000 °C sintered; (**g**) 1050 °C sintered; (**h**) 1100 °C sintered.

The physical parameters of the studied ceramic samples, including k_p_, Q_m_, d_33_, d_33_^*^, E_c_, and P_r_, are summarized in Table 2. The piezoceramics sintered at 980 °C have the optimal piezoelectric characteristics with a d_33_ of 701 pC/N and a k_p_ of 66.7%. Q_m_ values of all the samples remain constant at about 70, independent of sintering temperatures. Lead vacancies which are widely thought will result in low Q_m_, making the ceramics “soft”. Lead vacancies are propitious to domain switching. Thus, soft piezoelectric ceramics feature quick response speed, high piezoelectric constant, and low mechanical quality factor value. As reported, it is hard to ensure that the ceramics sintered at low temperatures have a high Q_m_ [41]. At low sintering temperatures, the effect of temperature on lead/oxygen vacancies is far less than that of doping [42]. This should be responsible for the temperature independence for Q_m_. The impedance plots are shown in Figure 4.

**Figure 5:**
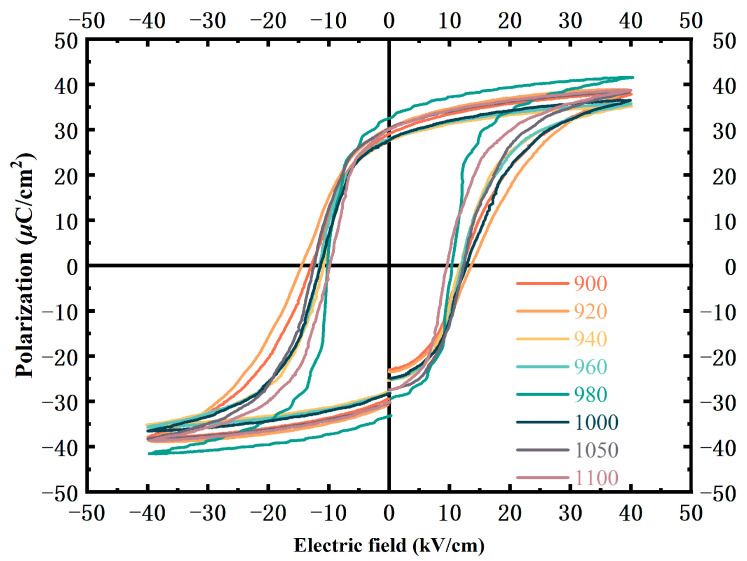
Ferroelectric hysteresis loop under 40 kV/cm.

Figure 5 illustrates the ferroelectric properties of the 0.2 wt% Li_2_CO_3_-modified ceramics. To achieve polarization saturation, the specimens were tested under 40 kV/cm, which is about four times E_c_. The maximum remnant polarization P_r_ (32.60 μC/cm^2^) can be obtained for the ceramics sintered at 980 °C. The remnant polarization has almost the same trend as the d_33_ due to the relationship of ε_r_P_r_.

As mentioned above, the piezoelectricity of the 0.2 wt% Li_2_CO_3_-modified ceramics exhibits slight variation with the increase in sintering temperature. Ultrahigh piezoelectric properties can be obtained under a low sintering temperature below 1000 °C, facilitating the use of cheap metal in the cofired ceramic devices.

**Figure 6:**
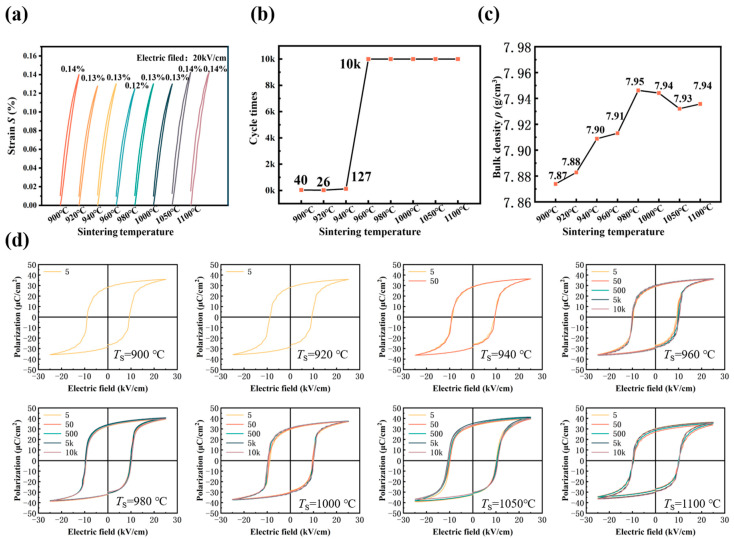
(**a**) Unipolar strain (*S-E*) curves of the 0.2 wt% Li_2_CO_3_-modified ceramics under 20 kV/cm; (**b**) The maximum cycle times; (**c**) Density of the 0.2 wt% Li_2_CO_3_-modified ceramics; (**d**) Cyclically *P-E* curve of piezoelectric ceramics under 25 kV/cm.

Figure 6a depicts the unipolar strain curves (*S-E*) of the 0.2 wt% Li_2_CO_3_-modified ceramics under 20 kV/cm. The strain of the ceramics remains almost constant on the variation of sintering temperature in the range of 0.12–0.14%. The normalized strain large-signal longitudinal piezoelectric constant d_33_^*^ can be calculated by Equation (3) and listed in Table 2.

(3) $$d_{33}^{*}=\frac{S_{\max}}{E_{\max}}$$

Regardless of the difference in grain size, piezoelectric strain remains constant, indicative of the maximization of reversible 90° domain switching.

However, piezoelectric ceramics are very brittle and susceptible to fracture during the electric drive. The electric field applied to the ceramic commonly causes the volume expansion/shrinkage of the unit cell of the ceramics. The variation of lattice volume usually causes internal stress, easily leading to the crack of piezoceramics. Cyclic testing of unpoled piezoelectric ceramics effectively verifies the fatigue behavior of piezoelectric devices [43]. Here, a working electric field of 25 kV/cm was selected to determine the cyclic fatigue properties by *P-E* loops of piezoelectric ceramics in Figure 6b. The ceramic samples were tested by 10^4^ times at 1 Hz with a triangle input signal. It can be observed that piezoelectric ceramics sintered at low temperatures become easy to dielectric breakdown, while the piezoelectric ceramics sintered above 960 °C can withstand 10^4^ cycles of electric excitation. It suggests that a high sintering temperature for ceramics contributes to the enhancement of fracture strength, possibly due to the improved bulk density or less porosity. It can be confirmed that the optimal *T*_s_ is near 960 °C, matching the sinterability of silver paste.

## 4. Conclusions

In this work, the 0.2 wt% Li_2_CO_3_-modified piezoelectric ceramics were developed to reduce sintering temperatures for low-temperature cofired applications. The influence of T_s_ on microstructure, dielectric, and piezoelectric properties was studied. Due to sintering aids, the piezoelectric ceramics could be densified at a low sintering temperature above 940 °C, suitable for co-firing with cheap electrodes. The piezoelectric ceramics can be optimized at a sintering temperature of 980 °C, accompanied by high piezoelectric d_33_ ~ 701 pC/N, k_p_ ~ 66.7%, and a low Q_m_ ~ 71 with a transition temperature of T_c_ ~ 226 °C, presenting the characteristics of typical soft piezoelectric ceramics. It had a promising strain of 0.13% under a low electric field (20 kV/cm) and was competitive for the fabrication of multilayer piezoelectric transducers.

## Figures and Tables

**Table 1:** Comparison of piezoelectric properties of PZT-based ceramics with sintering aids (*T*_s_: sintering temperature; *T*_c_: Curie temperature).

Sintering Aid	Ceramic Composition	*T*_s_ (°C)	*T*_c_ (°C)	*d*_33_ (pC/N)
Li_2_CO_3_ (0.07 wt.%) + Fe_2_O_3_ (0.25 wt.%) [30]	PZT-PZN-PMnN	1000		455
Li_2_CO_3_ (0.2 wt%) [31]	PNN-PZT	950	113	692
Li_2_CO_3_ (0.3 wt%) + Sm_2_O_3_ (0.3 wt%) [32]	0.3PZN-0.7PZT	900	394	483
Li_2_CO_3_ (0.2 wt%) + CaCO_3_ (0.3 wt%) [33]	PNN-PMW-PZT	900	296	599
Li_2_CO_3_ (0.2 wt%) + CaCO_3_ (0.25 wt%) + Ta_2_O_5_ (0.7 wt%) [34]	PNN-PMW-PZT	940	640	303
Yb_2_O_3_ (0.1 mol%) [35]	PNN-PMW-PZT	900	179	623
Sm_2_O_3_ (0.3 wt%) + LiF (0.01%) [17]	PZN-PZT	950	289	403
Ba(Cu_1/2_W_1/2_)O_3_ (0.1 wt%) [36]	PNN–PMW–PSN–PZT	900	161	551

**Table 2:** The specific values of electrical parameters of the 0.2 wt% Li_2_CO_3_-modified PZT-based ceramics.

T_sintering_ (°C)	T_c_ (°C)	d_33_ pC/N	d_33_^*^ pm/V	E_c_ kV/cm	P_r_ μC/cm^2^	k_p_ (%)	Q_m_
900	226	618	701	11.98	29.16	63.4	75
920	228	665	640	14.54	30.37	64.6	76
940	226	682	652	10.86	27.66	65.4	73
960	224	683	624	11.53	27.99	66.2	69
980	229	701	651	10.18	32.60	66.7	71
1000	229	661	648	11.28	27.68	66.3	70
1050	231	597	716	12.26	30.29	60.2	82
1100	226	591	721	10.03	30.12	58.1	78

## Data Availability

The datasets generated during and analyzed during the current study are available from the corresponding author on reasonable request.
